# Paediatric palliative care improves patient outcomes and reduces healthcare costs: evaluation of a home-based program

**DOI:** 10.1186/s12904-017-0267-z

**Published:** 2018-01-03

**Authors:** P. H. Chong, J. A. De Castro Molina, K. Teo, W. S. Tan

**Affiliations:** 1 HCA Hospice Care, Singapore, Singapore; 2 National Healthcare Group Singapore, Singapore, Singapore

**Keywords:** Palliative care, Paediatrics, Evaluation, Effectiveness, Costs, Caregiver burden, Health-related quality of life

## Abstract

**Background:**

Around the world, different models of paediatric palliative care have responded to the unique needs of children with life shortening conditions. However, research confirming their utility and impact is still lacking. This study compared patient-related outcomes and healthcare expenditures between those who received home-based paediatric palliative care and standard care. The quality of life and caregiver burden for patients receiving home-based paediatric palliative care were also tracked over the first year of enrolment to evaluate the service’s longitudinal impact.

**Method:**

A structured impact and cost evaluation of Singapore-based HCA Hospice Care’s Star PALS (Paediatric Advance Life Support) programme was conducted over a three-year period, employing both retrospective and prospective designs with two patient groups.

**Results:**

Compared to the control group (*n* = 67), patients receiving home-based paediatric palliative care (*n* = 71) spent more time at home than in hospital in the last year of life by 52 days (OR = 52.30, 95% CI: 25.44–79.17) with at least two fewer hospital admissions (OR = 2.46, 95% CI: 0.43–4.48); and were five times more likely to have an advance care plan formulated (OR = 5.51, 95% CI: 1.55–19.67). Medical costs incurred by this group were also considerably lower (by up to 87%). Moreover, both patients’ quality of life (in terms of pain and emotion), and caregiver burden showed improvement within the first year of enrolment into the programme.

**Discussion:**

Our findings suggest that home-based paediatric palliative care brings improved resource utilization and cost-savings for both patients and healthcare providers. More importantly, the lives of patients and their caregivers have improved, with terminally ill children and their caregivers being able to spend more quality time at home at the final stretch of the disease.

**Conclusions:**

The benefits of a community paediatric palliative care programme have been validated. Study findings can become key drivers when engaging service commissioners or even policy makers in appropriate settings.

## Background

Palliative care in children pursues similar goals as in the adult setting; to minimize suffering and to improve quality of life in the face of terminal illness [1, 2]. However, with great diversity in medical conditions and often uncertain trajectories, the context of a young person’s life unnaturally shortened by medical illness presents different needs and circumstances [3–7]. The World Health Organization has taken this uniqueness into consideration in their definition of Paediatric Palliative Care (PPC); specifically involving the family in healthcare decisions, using available community resources, and placing home as an option for the locus of care [8].

Overt moral imperatives and inherent benefits notwithstanding, PPC has yet to mature into a robust system of care that adequately meets the myriad needs of dying children and their families [9–13]. The Global Atlas of Palliative Care at the End of Life estimated that almost 1.2 million children between the ages of 0–14 years require end of life care [14]. However, according to Knapp et al. [15], two thirds of countries around the world still have no known PPC service provision at the time of their systematic review. Several factors have been postulated to contribute to PPC lagging behind its adult counterpart, including a dearth in evidence on the impact of care [16–18]. For example, benefits related to innovative service models in PPC that responded to local needs in different centres often lack validation in controlled studies [19]. In contrast, a rich corpus of literature in adult palliative care to demonstrate service impact has led to greater acknowledgement of its priority, which fostered wide integration within standard of care. Without critical appraisal of PPC through research, policy makers, healthcare providers and even the community at large will continue to have little confidence in its science and utility.

Recognizing the need for evidence-driven practice, various groups have identified research priorities within PPC [18, 20, 21]. Unsurprisingly for this young and developing subspecialty, effective interventions and models of care for PPC have regularly been flagged. Previous studies of different service models had mostly been descriptive in nature [22–24], an exception being Conte et al. [4], who investigated the effect of PPC programmes on resource utilization and costs in a systematic review. Summative results were mixed. Among patients who had received PPC, there was a drop in the proportion of patients with hospital admissions and a decreasing trend in average lengths of stay (LOS). However, comparisons between-groups (received PPC and controls) revealed no difference in number of hospital admissions, emergency room and outpatient visits. Analysis of healthcare cost differentials had also revealed conflicting findings.

Building on what have been found to date, a longitudinal evaluation of a new home-based PPC programme was performed.

## Objectives

Four specific questions were conceived and interrogated:

- Q1. In their final year of life, was there a difference in patient outcomes between paediatric patients under PPC, compared to equivalent comparators who received standard care? Outcomes tracked were (i) Proportion of time spent at home versus hospital; (ii) Proportion of patients who had Advance Care Plan (ACP) discussions; (iii) Number of Emergency Department (ED) visits, and (iv) Number of unplanned hospital admissions.
- Q2. In their final year of life, was there a difference in healthcare expenditure between patients who received PPC and those on standard care?
- Q3. Was there a change in patient’s Quality of Life after enrolment in the PPC programme?
- Q4. Was there a change in Caregiver Burden after enrolment in the PPC programme?

## Method

### Setting

Star PALS (Paediatric Advance Life Support) is Singapore’s first specialist home-based palliative care service that supports children less than nineteen years of age with life-shortening illness. The PPC programme began in 2012, with referrals coming predominantly from National University Hospital (NUH) or KK Women’s and Children’s Hospital (KKH), two government hospitals’ paediatric departments where most of the country’s sickest children are cared for. Star PALS is nested within an adult home hospice service (HCA Hospice Care) leveraging on existing structure and processes in its operations, such as electronic medical records and 24/7 helpline support. The team consists of healthcare professionals from various disciplines, including one specialist-grade physician, four paediatric nurses, two medical social workers, and one administrative executive.

### Design

A three-year cohort study (2012–2015) was carried out. A retrospective design was employed to answer evaluation questions Q1 and Q2. The cohort in this first phase consisted of two groups of deceased patients: those enrolled in Star PALS (PPC group) and those who were not enrolled in the programme (control group) and had died in hospital. A single-group prospective design for patients who had received PPC at home was employed to address questions Q3 and Q4 in a second phase.

### Inclusion / Exclusion criteria

Patients in both phases included only patients from NUH and KKH. Patients were included in the PPC groups if they were less than 19 years of age at the time of diagnosis, and received home-based palliative care. Otherwise, all children recruited had been diagnosed with a life-shortening condition that made them unlikely to survive into adulthood. The four categories of life limiting and life-threatening conditions from Together for Short Lives were referenced for diagnoses that were deemed life-shortening [25]. The criteria include:

i. Category 1: Life-threatening conditions for which curative treatment can fail.

(e.g. cancer, irreversible organ failures of heart, liver, kidney)

ii. Category 2: Conditions in which premature death is inevitable.

(e.g. Duchenne muscular dystrophy, cystic fibrosis)

iii. Category 3: Progressive conditions without curative treatment options.

(e.g. Batten disease, mucopolysaccharidoses)

iv. Category 4: Irreversible but non-progressive conditions causing severe disability, susceptibility to health complications and likelihood of premature death.

(e.g. cerebral palsy, multiple disabilities)

Patients were excluded from the study if they were neonatal cases, i.e. had survived less than 30 days after birth, as neonates were rarely referred for PPC locally at the time of the study. Patients with missing medical records from either hospital were also excluded, even if they had died there. Similarly, patients that died in a location other than home or hospital were excluded due to their medical data being incomplete or inaccessible.

### Method

Ethics approval was obtained for the full study from NUH and KKH, and relevant clinical and financial data were extracted from institutional administrative databases. Verification of patient eligibility was performed by two collaborating physicians, each affiliated with NUH and KKH respectively.

For the retrospective cohort study, the relevant data was extracted from medical records for coding and analysis:

i. Individual admission and discharge dates, for number of hospital admissions;
ii. Time of death, for cumulative length of stay;
iii. Emergency Department (ED) visits, for number of ED visits, and;
iv. Documentation or records of advance care planning (ACP) discussions that were held with family.

For the single-group prospective cohort study, the Health-Related Quality of Life (HRQL) of patients enrolled in the PPC group was assessed at 0, 3, 6, and 12 months using the Health Utilities Index (HUI). The HUI consists of two complementary health status classification systems – HUI2 and HUI3, assessing six and eight health-related attributes respectively [26]. Both health status classifications that make up the HUI are applicable across ages from five years and above, in both clinical and general populations [26]. Surveys were conducted through face-to-face interviews by a research associate, in either English or Mandarin. For patients below 12 years old and other patients with difficulty communicating or had cognitive impairment, parents acted as proxy respondents.

Similarly, caregiver burden was assessed serially within the same group prospectively at 0, 3, 6, and 12 months. The assessments were completed with the main caregiver for each child using Zarit Burden Interview (ZBI) – a face-to-face, 22-item instrument [27]. It contains statements which reflect how people feel when taking care of another person. Each response corresponds to a number in points. Scores for each statement are then summed for a total score from 0 to 88, with higher scores indicating more severe burden. The interview has been assessed to have sound psychometric properties and is widely used across languages and culture [27]. While the tool was originally intended for caregivers of elderly patients with dementia, it has been used for caregivers of paediatric patients as well [28].

### Sample size

Using available historical figures from Star PALS over the 3-year study period, 150 patients were expected to be enrolled to receive palliative care at home, with an average mortality rate of 33%. With the anticipated sample size of 50 deceased PPC patients and 50 deceased controls, an α error of 0.05 for a 2-sided test, and PPC patients spending 60% in the last year of their life at home instead of the hospital (based on previous figures), the estimated power for the study to detect a 10% difference between groups was 70%.

### Statistical analyses

Q1. Mean differences between the PPC group and control group for (i) Number of hospitalizations up to 1 year before death, (ii) 1-year cumulative hospital length of stay (LOS), (iii) Proportion of time spent at home, and (iv) ED visits, along with their corresponding 95% confidence intervals for any differences, were generated. In addition, the odds ratio for having an ACP was estimated.

Q2. Economic impact in this study refers to the dollar value of health resources consumed by patients at the end of life [29, 30]. The costing analysis was conducted from the healthcare system perspective, and included both healthcare and intervention costs.

Healthcare costs refer to the cost of healthcare resources utilized, including hospitalization, ED visits and outpatient visits. The total medical bill before any deduction for government subsidies and insurance claims, were used to estimate healthcare resources consumed [31].

For the PPC group, on top of healthcare costs, additional costs of PPC services (intervention costs) were ascertained using fixed and variable costs of the Star PALS programme, according to Star PALS’s financial statements.

Total cost-per-patient in the PPC group was derived by the sum of fixed-cost-per-patient and variable-cost-per-patient. Fixed cost-per-patient was derived using the programme’s total fixed costs divided by the total number of patients served over a period of three years. Variable costs included visits to patients’ home or hospital by physicians, nurses, medical social workers, allied health professionals, and respite caregivers. Per-visit costs were estimated using total manpower expenditure divided by the total number of visits, or the provider fee per visit.

Total medical costs incurred by PPC group were compared with the control group at the following time points: 12 months (360 days), 6 months (180 days), 3 months (90 days), and 1 month (30 days) prior to death. Data between groups were compared using Chi-Square (χ^2^) tests for categorical variables and Wilcoxon Rank Sum test for abnormally distributed continuous variables. Univariate analyses (ANOVA) were conducted for both primary and secondary outcomes. Multivariate analyses (MANOVA) adjusted for baseline differences at *p* < 0.05 were performed for the primary outcomes. Variables statistically significant at *p* < 0.05 were included in the multivariable generalized linear model (GLM) with log link function and gamma family. The model was used to compare skewed hospital LOS and healthcare expenditure. The method of recycled predictions was used to obtain predicted mean costs of both groups [32].

Q3. Point estimates for the change from baseline to each period of follow-up for every domain of HUI were generated.

Q4. Point estimates for differences between ZBI scores at baseline and each period of follow-up, as well as the 95% confidence intervals for each difference were generated.

## Results

For the first phase involving a retrospective cohort, 79 deceased patients were identified in the PPC group; 8 patients were excluded, resulting in a total of 71 patients. Among 348 other patients who died in the two hospitals over the same period, 233 patients were excluded as they did not fit disease criteria for PPC, and 48 were excluded as they were neonatal cases, resulting in 67 patients being recruited in the control group, having never received PPC. The demographics and other characteristics between both groups were compared (Table 1).

**Table 1:** Comparison of baseline characteristics

Profile	PPC group (*n* = 71)	control group (*n* = 67)	*p*
Age at death (years)^a^			
Mean (SD)	12.2 (6.9)	6.3 (6.3)	.000*
Gender^b^			
Male, no. (%)	45 (63.4)	41 (61.2)	
Female, no. (%)	26 (36.6)	26 (38.8)	.079
Ethnicity^b^			
Chinese (%)	41 (58.6)	35 (52.2)	.037*
Malay (%)	24 (34.3)	16 (23.9)	
Indian (%)	2 (2.9)	3 (4.5)	
Others (%)	3 (4.3)	13 (19.4)	
Residency Status^b^			
Singapore Resident (%)	35 (49.3)	9 (13.4)	.000*
Non-Resident (%)	36 (50.7)	58 (86.6)	
Referral Source^b^			
KKH	34 (47.9)	44 (65.7)	.011*
NUH	30 (42.3)	23 (34.3)	
Others	7 (9.9)	0 (0.0)	
Cause of Death^b^			
Neoplasms	32 (45.1)	28 (41.8)	.698
Others	39 (54.9)	39 (58.2)	

*p < .05; ^a^t-test; ^b^Chi-Square test

The distributions of cancer and non-cancer cases were similar between groups, but the control group had significantly younger patients and more non-residents (*p* < .05).

### Comparison of patient outcomes and healthcare utilization

Table 2 shows the comparison of key outcomes between the PPC group and the control group over a full-year of follow-up until death. First, those in the PPC group (M = 4.47, SD = 4.58) had 2.46 fewer mean hospital admissions (95% CI: 0.43–4.48) than the control group (M = 6.93, SD = 5.51). Moreover, the mean 1-year cumulative length of stay (LOS) in hospital for PPC group (M = 40.79, SD = 50.97) was lower than the control group (M = 93.1, SD = 77.38) by 52 days (95% CI: 25.44–79.17). On the other hand, there was no significant difference in the mean number of ED visits between PPC group and the control group over 12 months (*p* > .05*)*.

**Table 2:** Comparison of outcomes between groups

	PPC group		control group		Mean difference
	N	Mean (SD)	N	Mean (SD)	(95% CI)
Hospital admissions over 12 months	68	4.47 (4.58)	42	6.93 (5.51)	2.46 (0.43, 4.48)*
1-year cumulative length-of-stay (LOS)	68	40.79 (50.97)	42	93.1 (77.38)	52.30 (25.44, 79.17)*
Emergency Department visits over 12 months	68	1.99 (2.87)	43	1.81 (2.23)	−0.17 (−1.19, 0.85)

*p < .05

Additionally, patients in the PPC group were approximately 5 times more likely (OR = 5.51, 95% CI: 1.55–19.67) to have an ACP, or to have discussed ACP with a healthcare professional, compared to the control group.

### Economic impact of healthcare utilization

**Fig. 1:**
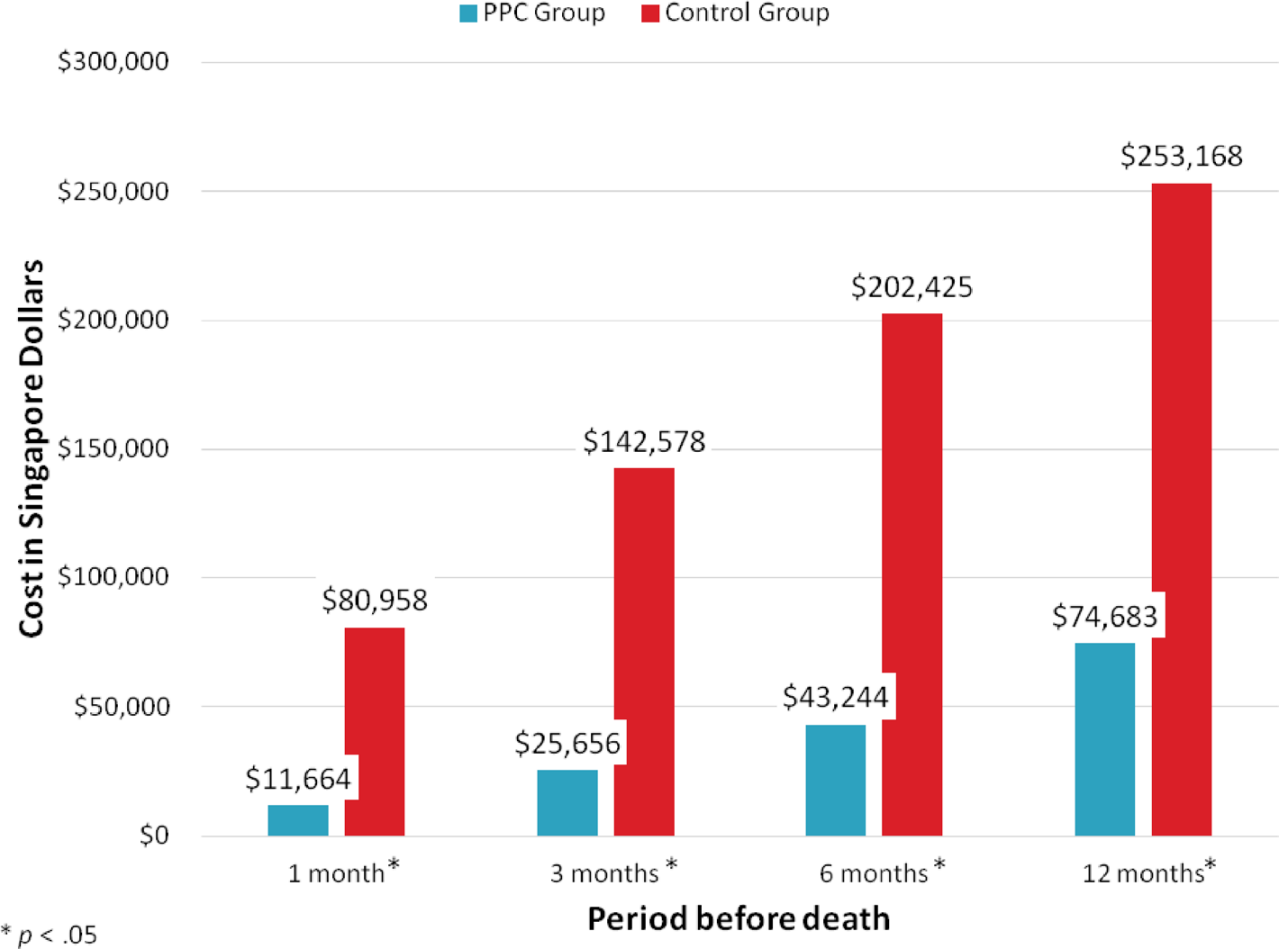
Comparisons of differences in medical costs between groups

Figure 1 shows the univariate analysis of unadjusted medical costs. Cost of care for control group at the end of life was SGD $253,168 per year, with 32% (SGD$80,958) incurred within the final month of life. Cost of care per year was significantly lower (SGD$74,683) in the PPC group (*p* < .05).

Table 3 illustrates the mean cost savings after adjusting for significant differences in their baseline characteristics (namely age, ethnicity, residency status, and referral source). At 12 months prior to death, medical costs for PPC group were 70% (SGD$ 175 K) lower than the control group. Cost savings increased to 87% (SGD$ 72 K) at one month prior to death.

**Table 3:** Multivariate analysis of adjusted^a^ medical costs between groups

Months prior to death	Rate Ratio (CI)	Mean incremental costs
12-month	0.30 (0.20, 0.45)*	-$175 k (−$186 k, −$164 k)
6-month	0.22 (0.16, 0.33)*	-$151 k (−$159 k, −$143 k)
3-month	0.18 (0.13, 0.25)*	-$116 k (−$121 k, −$111 k)
1-month	0.13 (0.09, 0.18)*	-$ 72 k (−$ 76 k, −$ 69 k)

^a^Adjusted for age, ethnicity, residency status, and referral source

Reference: control group

*p < .05

### Prospective evaluation on patient quality of life and caregiver burden

For the prospective analysis of HRQL, 56 respondents who received PPC were included. Table 4 shows their demographic profile. One caregiver for each of the 56 patients was also surveyed. The mean age of the caregivers was 39.8 years (*SD* = 6.4), with 81% female.

**Table 4:** Profile of patients (*N* = 56)

Profile	Units
No. with 1 follow-up (at 3 months before death)	42
No. with 2 follow-ups (3- and 6 months before death)	26
No with 3 follow-ups (3-, 6-, and 12 months before death)	10
Mean Age (SD)	8.45 (6.12)
Age 0–4, no. (%)	20 (35.71)
Age 5–12, no. (%)	17 (30.36)
Age 13+, no. (%)	19 (33.93)
Gender	
Male, no. (%)	23 (41.1)
Female, no. (%)	33 (58.9)
Primary Diagnosis	
Congenital Defect, no. (%)	28 (50.0)
Neoplasm (including Leukaemia), no. (%)	10 (17.8)
Cerebral Palsy, no. (%)	7 (12.5)
Neurodegenerative disorders, no. (%)	8 (14.3)
Others, no. (%)	3 (5.4)

### Health-related quality of life of patients over time after receiving PPC

Point estimates suggest that, compared to baseline, a greater proportion of patients had unchanged or more severe levels of sensation, mobility, cognition and self-care (based on HUI2), as well as vision, speech, ambulation, dexterity and cognition (based on HUI3), on all periods of follow-ups. However, a greater proportion was reported to have improved emotion and reduced pain on follow-up. Tests of significance were conducted to confirm these observations. The odds of being pain-free at 3 months (OR = 2.58, 95% CI: 1.12–5.95) were significantly higher than at baseline (*p* < 0.05).

### Caregiver burden over time after receiving PPC

Table 5 shows the mean ZBI scores and point estimates from the respondents at baseline (i.e. when service was first provided) and the three periods after baseline. During all four periods, caregivers indicated mild to moderate burden. Point estimates showed a decrease in scores from baseline up to 6 months, followed by an increase at 12 months. Scores at 3 months (OR = 3.4, 95% CI: 1.0–5.9) and 6 months (OR = 4.0, 95% CI: 0.5–7.5) were found to be significantly lower than at baseline (*p* < 0.05).

**Table 5:** Zarit Burden Interview Mean scores and differences

	Baseline *N* = 56	3 months from baseline *N* = 42	6 months from baseline *N* = 26	12 months from baseline *N* = 7
Mean Score	33.9	31.0	29.9	38.7
95% CI of the mean score	30.5, 37.3	27.2, 34.8	24.7, 35.1	30.6, 46.9
Mean difference compared to baseline (95% CI)		3.4 (1.0, 5.9)*	4.0 (0.5, 7.5)*	0.9 (−11.0, 12.7)

*p < 0.05

## Discussion

To our knowledge, this is the first evaluation of a PPC model of this scale from within the Asia Pacific region. Given the influence of context and healthcare financing on service uptake and satisfaction, findings from this study can inform both practitioners and policy makers in neighbouring settings that may share common characteristics [18]. Critically, this report adds to the growing evidence globally on best practices in the care of children with life-shortening conditions that respect the tenet of family-centred care, especially within the home setting [33].

Compared to patients who had not received PPC, patients under the home-based PPC programme had fewer and shorter hospital admissions, allowing them to spend more time at home in their last year of life. In comparison, a study in an established home-based PPC service in the United States did not show any change in the average total number of admissions pre- and post- PPC, but was able to reduce LOS and total charges for hospital-based care in patients with non-cancer diagnoses. Interestingly, the study found an increase in hospital emergency room admissions among children with cancer diagnoses over time [34]. In our study, visits to the ED did not show any significant difference between groups except in the quarter just before death.

This study found evidence for improved quality of life in the domains of pain and emotion among patients. The same cohort also revealed reduced caregiver burdens at 3 and 6 months after enrolment into the PPC programme. Similarly, Groh et al. [35] in Germany reported significant improvement in both children’s symptoms and quality of life with specialized paediatric palliative home care. The investigators in that single-group prospective study also found significant reduction in caregiver burden, psychological distress and quality of life. Follow-up of all respondents in that study was over a median of only 8 weeks though.

Kaye et al. [10] had commented that aggressive and high-technology care that may not be in the best interest of the dying child remains a major cost driver in today’s healthcare model. The extensive support rendered to patients and families throughout the disease trajectory and at end of life, coupled with advance care plans that limited life sustaining therapy just before death, likely led to reductions in healthcare expenditure within the PPC group. The cost of providing such a service to one child over a year was SGD$9568 (in intervention cost). As Star PALS operates within an established adult home hospice with existing structures and processes, incidental costs from other departments like human resource or finance, and to provide 24/7 helpline support were not considered within the controlled study. If they were all included, the estimated cost of care for one child a year goes up to SGD$13,363. In a novel multi-method study by Noyes et al. in the United Kingdom, the cost for supporting a similar child with PPC was found to be comparable at SGD$3002 - SGD$13,606 (£2437 – £11,045) [36]. However, the same authors also found that just to care for one dying child at home for an extra week would cost SGD$17,246 (£14,000) more, highlighting inherent challenges of sustaining a community-based service in some settings. Another group in the United States documented a 32% reduction in hospitalisation days after providing in-home community-based PPC, saving US$1677 per child per month (an 11% decrease) [37]. A control group in that study might have shown equally hefty cost savings like ours, within their highly advanced medical setting.

The study design in this evaluation has some limitations. Firstly, the control group consisted only of patients with life-shortening conditions that had died in hospital. This study’s goal was to compare the impact of home-based PPC with that of standard care. It remains today that most children with life-shortening conditions die in the hospital [38, 39]. Furthermore, accurate clinical and financial data in the control group particularly near the end of life was available only for hospitalised patients.

Second, there were differences in some baseline characteristics between both groups that needed consideration during data analysis, namely age, ethnicity and residential status. These factors could have introduced bias, particularly with respect to utilization of health services. We controlled for these factors in our adjusted analyses of cost savings.

Third, a comparative prospective design for HRQL and Caregiver Burden was not feasible as it was deemed unethical to withhold PPC in one group for study completion. Threats to validity including maturation and historical effects therefore cannot be discounted.

Moreover, for the retrospective cohort component, it was not possible to extract data for eight patients in the PPC group, and they thus had to be excluded (two had missing records and six others had died in an adult hospital, a hospice or outside the country).

It is also acknowledged that the healthcare system and socio-cultural context within which the study was conducted may limit the generalisability of findings shared here.

Hence, future research can focus on assessment of quality of care in the implementation of care models across diverse settings [40], to establish consistent delivery of putative benefits. With studies in the adult setting showing modest benefits of increased survival after early palliative care, it would be appropriate to include survival as an additional outcome measure in longitudinal studies of children receiving PPC. 

## Conclusion

The cost of PPC; interventions and models of care; and measuring outcomes of care are all listed within top ten priorities for global research in children palliative care [21]. This mixed closed-cohort study has not only uncovered the cost of providing this specialized care at home, it has demonstrated optimization of resource utilization, with concomitant healthcare cost savings. More importantly, the lives of children and their caregivers have improved in the process.

## Abbreviations

ACP: Advance Care Plan
ED: Emergency Department
HRQL: Health Related Quality of Life
HUI: Health Utilities Index
KKH: KK Women’s and Children’s Hospital
LOS: Lengths of Stay
MSW: Medical Social Worker
NUH: National University Hospital
PALS: Paediatric Advance Life Support
PPC: Paediatric Palliative Care
ZBI: Zarit Burden Interview
